# Chemophysical Characterization of Carotenoproteins From Crustacean Shell Waste for Pharmaceutical Applications

**DOI:** 10.1155/ijfo/1042650

**Published:** 2026-06-15

**Authors:** Luisa Di Paola, Gabriella Caruso, Francesca Manganiello, Chiara Bianca Maria Platania, Adriana Albini, Douglas Noonan, Claudio Serangeli

**Affiliations:** ^1^ Departmental Faculty of Engineering, Università Campus Bio-Medico di Roma, Rome, Italy, unicampus.it; ^2^ Institute of Polar Sciences, National Research Council, Messina, Italy, cnr.it; ^3^ Department of Biomedical and Biotechnological Sciences, School of Medicine, University of Catania, Catania, Italy, unict.it; ^4^ Center for Research in Ocular Pharmacology (CERFO), University of Catania, Catania, Italy, unict.it; ^5^ Scientific Directorate, European Institute of Oncology (IEO), IRCCS, Milan, Italy, ircc.it; ^6^ Department of Biotechnology and Life Sciences, University of Insubria, Varese, Italy, uninsubria.eu; ^7^ AGC 98 S.r.l., Rome, Italy

**Keywords:** astaxanthin, Atlantic blue crab, biorefinery, *Callinectes sapidus*, crustacyanin, European lobster, *Homarus gammarus*, Keap1

## Abstract

Commercial crustacean species represent some of the most economically valuable fishery products; however, their full potential remains underexploited due to the routine disposal of shellfish waste, which is rich in high‐value bioactive compounds. Among these, carotenoid pigments have garnered increasing interest for their antioxidant and anti‐inflammatory properties. In this study, we investigate the potential of recovering bioactive compounds from the shells of the European lobster (*Homarus gammarus* Linnaeus, 1758) and the Atlantic blue crab (*Callinectes sapidus* Rathbun, 1896), with a particular focus on carotenoprotein complexes with promising applications in the pharmaceutical and cosmetic industries. We adopted a computational approach integrating protein contact networks, molecular docking, and molecular dynamics simulations to characterize the structural and functional properties of crustacean carotenoproteins as nanocarriers for astaxanthin. Then, we explored in silico the antioxidant activity of astaxanthin, as claimed by many studies. Specifically, we applied the computational analyses to the interaction between astaxanthin and Kelch‐like ECH‐associated protein 1 (Keap1‐DC domain), a key regulator of the Nrf2 signaling pathway involved in oxidative stress. Results suggest that astaxanthin would compete with Nrf2 at the Keap1‐DC domain, then promote Nrf2 induction and activate the antioxidant cellular machinery. These findings are supported by preliminary experimental evidence and highlight the potential of astaxanthin extracted from crustacean shell waste as a bioactive agent, with possible applications in diseases associated with oxidative stress, including cancer. Further preclinical and clinical in vivo studies are needed to validate astaxanthin and the carotenoprotein–astaxanthin complex for efficacy and safety, with potential relevance for oncology.

## 1. Introduction

The seafood processing industry generates 50%–80% of waste by weight [1], produced by fishing and farming activities. Seafood waste refers to both edible and inedible raw materials that are discarded across the food chain, with consequent losses deriving from practices adopted by producers, retailers, food service operators, and consumers [2].

Crustaceans—including shrimp, lobster, crab, and crayfish—are among the most economically important species in the global seafood industry, representing approximately 5%–10% of total seafood production volume [3, 4]; their high market prices further amplify their share in terms of overall market value.

Crustacean processing (especially of crab and lobster) generates as much as 30%–85% solid waste, including shells and heads [2, 5, 6], depending on the species and on the processing method, handling procedures, and storage conditions [7, 8]. Crustacean shell waste alone generates an estimated 6–8 million tons annually, thus being one of the largest streams of by‐products arising from seafood processing [9].

The biorefinery approach allows for transforming seafood processing waste and by‐products into value‐added products, thereby addressing environmental concerns while complying with circular economy principles [10]. In this scenario, crustacean wastes offer a unique opportunity for the sustainable valorization of products with high economic potential, which would otherwise be lost.

In this work, we focus on by‐products from two decapod crustacean species: the European lobster (*Homarus gammarus* Linnaeus, 1758) and the Atlantic blue crab (*Callinectes sapidus* Rathbun, 1896).

*H. gammarus* is a commercially appreciated lobster species widely distributed in the European region. Its distribution in the benthic ecosystem is controlled bottom‐up by food availability and top‐down by predation [11]. Lobster processing generates a high amount of waste (heads, shells, eggs, and livers; over 50,000 tons annually [12]), covering 50%–70% of the total shellfish waste for all commercial species.

The valorization of lobster processing waste is highly recommended thanks to the presence of several high‐value compounds such as proteins, chitin, lipids, minerals, and pigments with nutraceutical, cosmetic, and pharmaceutical applications.

Likewise, the shell waste from *C. sapidus* is a resource that deserves to be exploited for its biotechnological potential. *C. sapidus* is an invasive species in the Mediterranean region, representing an emerging ecological and socioeconomic threat [13]. Due to its opportunistic predation behavior on fish eggs, mollusks, crustaceans, and benthic invertebrates, this species disrupts native food webs and reduces biodiversity, causing disturbances to sediment structure and aquatic vegetation and affecting nursery habitats for native species. Due to its competition for food and space with native crabs and other benthic organisms, *C. sapidus* can cause significant shifts in local communities and habitat alteration. The species’ high reproductive rate and adaptive ability to colonize estuaries, lagoons, and coastal waters make the containment of the spread of *C. sapidus* increasingly difficult in temperate environments.

*C. sapidus* negatively impacts both tourism and fisheries, directly threatening the economy of coastal communities. It is therefore necessary to implement effective strategies for the monitoring and control of this invasive species. One viable route is to valorize *C. sapidus* catches beyond their use in the food sector, fully exploiting the species’ economic potential [14]. This approach is sustainable in the long term, as it simultaneously supports population control and strengthens the economy of coastal communities, enhancing their resilience to environmental pressures such as climate change and the growing presence of invasive species.

Recent advances in biorefinery processes have underscored the importance of sustainably extracting value‐added compounds from biological feedstocks to improve the resource efficiency of industrial processes in the pharmaceutical, nutraceutical, and cosmetic fields [15]. In the fishery sector, the biorefinery approach aims at valorizing all components of fishery products to produce additional value and reduce the volume of production waste to near zero [16]. This approach is particularly impactful if applied to residual biological sources and production waste within the circular bioeconomy framework [8, 17].

In this work, we explore the structural and functional properties of the carotenoproteins present in the shells of *H. gammarus* and *C. sapidus.* We also evaluated the activity of astaxanthin, the carotenoid complexed and delivered by the decapod carotenoproteins, as a putative antioxidant agent, targeting Keap1.

For the analysis of protein molecular structures, we applied molecular dynamics (MD) and molecular docking to provide a quantitative structural analysis of the carotenoproteins from the two species.

To obtain a residue‐level characterization of the functional properties of the shell carotenoproteins from both species and of the human Keap1 forms—in their unbound state and in complex with astaxanthin—we employed the protein contact network (PCN) approach. This method has been extensively validated as a powerful framework for probing the structural and functional role of individual residues within protein architectures [18] and has proven particularly effective in elucidating mechanisms of allosteric regulation [19, 20].

We also explored in silico the activity of astaxanthin as a therapeutic agent toward oxidative stress by molecular docking against Keap1.

Astaxanthin is a potent antioxidant due to direct physical and chemical quenching of reactive oxygen species [21]. Beyond its direct radical‐scavenging capacity, astaxanthin exerts indirect antioxidant activity through the transcriptional activation of cytoprotective machinery, most notably the nuclear factor erythroid 2‐related factor 2/heme oxygenase‐1 (Nrf2/HO‐1) signaling pathway [22], which is also implicated in cancer development and response to therapy.

Therefore, based on the astaxanthin structure, we identified the kelch domain of Keap1 as a putative pharmacological target, which is a negative regulator of Nrf2 activity and a recognized molecular node in oxidative stress– and cancer‐related signaling. Any compound able to interfere with the Keap1–Nrf2 interaction is an Nrf2 inducer, thus promoting activation of the cellular antioxidant machinery [23–26]. Astaxanthin cannot induce Nrf2 through nucleophilic addition with Cys151 of the Keap1‐BTB domain [27], because it lacks an sp2 leaving group for any nucleophilic addition, such as Michael addition. Astaxanthin would likely be a protein–protein interaction disruptor by interfering with the interaction between the Keap1 kelch domain (Keap1‐DC) and Nrf2 [28].

In this study, we predict the binding of astaxanthin to the Keap1 kelch domain by molecular docking and analyze the Keap1–astaxanthin complex by means of MD simulations, comparing the results with those for the unbound Keap1 form.

## 2. Materials and Methods

### 2.1. Structural Information of Molecules

We introduced different computational methodologies to clarify the molecular function of shell carotenoproteins through quantitative descriptors.

**Figure 1:**
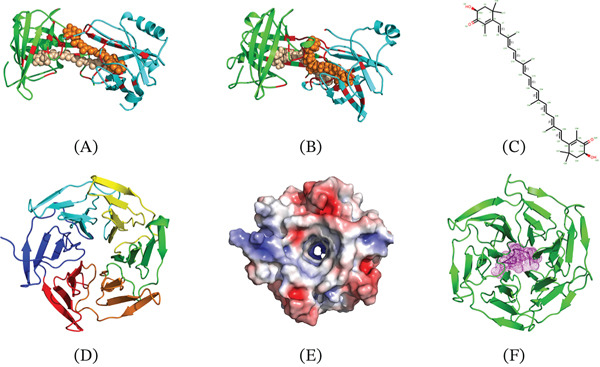
Molecular structures of interest. (A) *H. gammarus β*‐crustacyanin (PDB: 1GKA). (B) *C. sapidus* apolipoprotein D‐like protein (model built as reported in Section 2). In green (Chain A) and blue (Chain B) are the two chains binding two molecules of astaxanthin (yellow and orange spheres); the residues in contact with the two molecules of astaxanthin are highlighted in red. (C) Chemical structure of astaxanthin (3,3 ^′^‐dihydroxy‐*β*,*β*‐carotene‐4,4 ^′^‐dione). (D) Keap1 kelch domain structure: the six blades (domains) are shown in different colors. (E) Electrostatic surface potential representation of the kelch domain. Blue indicates negatively charged regions, red corresponds to positively charged areas, and white highlights hydrophobic surface patches. (F) Keap1 kelch domain bound to the Nrf2 peptide (in magenta mesh in the figure).

The computational methods were applied to the following molecular structures provided as PDB files:

– *β*‐Crustacyanin of *H. gammarus*, PDB code 1GKA [21]: the molecule is a heterodimer (see Figure 1A);
– Carotenoprotein of *C. sapidus*: the molecular structure shown in Figure 1B was designed by applying the following protocol: first, we identified the gene of the *C. sapidus* apolipoprotein D‐like protein (https://www.ncbi.nlm.nih.gov/protein/2091619298) by means of BLAST using as a template the crustacyanin A2 subunit of *H. gammarus* (P80007.1, Chain B in PDB: 1GKA). We did not find in *C. sapidus* any homologous protein to the crustacyanin A1 subunit of *H. gammarus* (Chain A in PDB: 1GKA), so we supposed that the biologically active form of *C. sapidus* is a homodimer. The monomer model for the *C. sapidus* apolipoprotein was created by means of the SwissModel platform (https://swissmodel.expasy.org/) using the abovementioned sequence and the structure of *H. gammarus* as a template; the dimer bound to two molecules of astaxanthin was then created in the PyMOL environment using *H. gammarus* crustacyanin as a guide for superposition. All structures underwent energy minimization before the MD simulation. In Figure 1, the two astaxanthin molecules are in the inner cavity of the two heterodimers, made up of two moieties belonging to the two chains. In *H. gammarus*, *β*‐crustacyanin is a heterodimer belonging to the lipocalin family [29], binding two astaxanthin molecules (orange and yellow, represented as VdW spheres in Figure 1A).

In the model of the *C. sapidus* complex, the two molecules of astaxanthin appear more twisted (see Figure 1B). Figure 1 also shows the residues in contact with the astaxanthin molecules (in red): the distribution of these residues is quite different in the two conformations, being more extended in the crustacyanin of *H. gammarus* than in the apolipoprotein of *C. sapidus.*

For both conformations, the distribution of residues in contact with astaxanthin is uneven: in *H. gammarus*, 33 residues in Chain A are in contact with astaxanthin and eight in Chain B, while in the *C. sapidus* complex, 32 residues are in contact with astaxanthin in Chain A and 21 in Chain B.

– Astaxanthin (its molecular structure is shown in Figure 1C): it has gained attention for its beneficial health effects, including its potential role in preventing oxidative stress–related diseases [21, 30]. Astaxanthin (3,3 ^′^‐dihydroxy‐*β*,*β*‐carotene‐4,4 ^′^‐dione) is a major pigment in decapod shells: it is a carotenoid xanthophyll pigment with the chemical formula C_40_H_52_O_4_, a molecular mass of 596.85 Da, and a density of 1.081 g/L (see Figure 1C).
– Keap1, the kelch domain of human Keap1, PDB code 1U6D [26]: it is shown in Figure 1D (sequence segment 322–609). The kelch domain of human Keap1 comprises six kelch repeats forming a symmetric, six‐bladed *β*‐propeller structure (50 Å in diameter and 35 Å in height, with a small channel—around 6 Å in diameter) through the center of the propeller. In Figure 1E, the narrowest part of the kelch domain is negatively charged and interacts directly with the LDEETGEFA peptide. Table 1 reports the sequence distribution of the six blades in different colors from blue to red in Figure 1E.

**Table 1:** Distribution along the sequence of the six blades of the kelch domain of Keap1.

Blades	Sequence
I	322–365; 597–609
II	366–416
III	417–463
IV	464–510
V	511–557
VI	558–596

Figure 1F shows the complex of Keap1 with the LDEETGEFA Nrf2 peptide (PDB code 7K2A): the binding site is on the narrowest side of the kelch domain, at the center of the propeller.

### 2.2. Computational Analysis of Molecular Structures

#### 2.2.1. MD and Molecular Docking Simulations

Classical MD simulations were performed by means of the open‐source software GROMACS v. 2024.4 [31] with the AMBER99SB‐ILDN force field [32]. We performed MD simulations of 1 ns for all structures described above on the Neurosnap platform (https://neurosnap.ai/); each simulation was replicated three times.

Each system was placed in a cubic box with periodic boundary conditions. The box size was automatically adjusted in each case to optimize the MD simulation. Each box was filled with water molecules and an appropriate number of counterions (Na^+^ and Cl^−^) necessary for system neutrality (0.15 M). The temperature was kept in the NPT ensemble at 300 K and the pressure at 1 bar.

We applied a canonical analysis of motion to the MD frames to identify concerted motions of protein regions, according to a procedure explained extensively elsewhere [33].

Briefly, once the displacement matrix (DISPL) is computed from the MD trajectory, it is possible to compute the dynamic cross‐correlation (DCC) matrix, whose generic elementDCC_*i* *j*_is the Pearson correlation coefficient between the displacement vectors of the *i*‐th and*j*‐th residues. The DCC allows one to highlight the concerted motions between distal residues [33]: high values of the DCC matrix highlight the high probability of concerted motions of residue pairs.

In this work, we introduced a correction to the displacement evaluation for each residue to get rid of effects due to rototranslation and to keep only motion contributions that are independent of rigid body movements of the whole molecular structure [34].

We performed molecular docking of astaxanthin with Keap1 by means of the DiffDock‐L molecular docking software [35] on the Neurosnap platform. The receptor–ligand complex was then annealed by means of MD simulations, as previously described.

#### 2.2.2. PCN Analysis

We applied the method of PCNs to analyze the protein molecular structures of carotenoproteins and both forms of the kelch domain of Keap1. For all structures, we analyzed the last frame after MD relaxation, as detailed above.

The PCN method is based on the representation of the protein molecular structure as a network, whose nodes are the single residues and whose links between nodes are the active noncovalent contacts (between residues separated by 4–8 Å) [36]. The network is described by means of an undirected unweighted graph, whose mathematical representation is provided by the adjacency matrix *A*, defined as follows:

(1) A=148 if  A°<dij< A°,0 otherwise.

The network is characterized by the following descriptors derived from the adjacency matrix:

– Node degree: it identifies for each node the number of links it participates in and is computed as follows:
(2) ki=∑JAij,

where the node degree in PCNs identifies the relevance of the node (residue) in network (structure) stability.

– Shortest path matrix: between the*i*th and*j*th residues, there exist many connecting paths across the network, with different path lengths; the shortest path sp_*i* *j*_ reports the path with minimum length (in terms of links to be traversed) among all paths connecting the *i*th and *j*th residues in the PCN. The relevance of the shortest paths in protein functionality is quite clear: the network of noncovalent interactions, adjusting to environmental stimuli, is also the backbone for signal transmission throughout the protein molecular structures. The shortest paths are then the “signal highways” through which the signal is swiftly transmitted.
– Betweenness centrality: tightly connected to the shortest path, the betweenness centrality is computed for each node and describes the number of shortest paths passing by it. The betweenness centrality in PCNs defines the relevance of each single node in the signal transmission through distal regions of the molecular structures.
– Eigenvector centrality: for the generic *i*th node, it is defined as follows:
(3) xi=∑JAijxj,

where the sum is extended to all nodes; this recursive definition can be easily solved as an eigenvector decomposition problem applied to the adjacency matrix *A*. According to the Perron–Frobenius theorem, the eigenvector corresponding to the maximum eigenvalue (principal eigenvector) has only positive values, and the components define the eigenvector centrality for each corresponding node [37].

The eigenvector centrality addresses the relevance of a node in terms of its direct interactions with highly connected nodes; nodes with high eigenvector centrality scores play a central role in the communication between PCN domains and chains in multichain proteins [38].

– Modularity: another central feature of proteins caught by PCNs is their modularity, well interpreted in terms of PCN clustering [39]. Indeed, it is possible to identify protein modules by means of PCN spectral clustering [40]. This method applies to the PCN Laplacian matrix *L* defined as follows:
(4) L=D−A,

where *A* is the adjacency matrix and *D* is the degree matrix (i.e., a diagonal matrix whose diagonal is the degree vector). The spectral decomposition of the Laplacian matrix *L* identifies the eigenvector corresponding to the second‐smallest eigenvalue *v*_2_, known as the Fiedler vector, used for the clustering partition.

Upon clustering, it is possible to compute the participation coefficient *P* defined for each residue as follows:

(5) Pi=1−ksiki2,

where *k*_*s* *i*_ is the number of links the *i*th node shares with nodes belonging to its own cluster. The participation coefficient quantifies the role of individual residues in mediating intercluster signal transmission. Values range from 0 to 1, where values approaching unity indicate a strong propensity of the node (residue) to facilitate communication across distinct protein modules.

We computed all PCN descriptors by means of PyPCN, a plugin for PyMOL [41]. We report results of the PCN descriptors (betweenness and eigenvector centralities, participation coefficient, and cluster partition) for all molecules listed in Section 2 as heat maps on ribbon structures. Heat maps have been built using a purpose‐built Python script in the PyMOL environment, reporting data from the PyPCN application.

We have also reported DCC heat maps to identify concerted motions between protein regions, with the maps produced in the MATLAB environment.

#### 2.2.3. General Biophysical and Interface Analysis

The general analysis of the protein molecular structure and of the protein–protein interface was performed using the PISA web server (https://www.ebi.ac.uk/pdbe/pisa/), deriving different thermodynamic parameters.

The following is a brief explanation of the major descriptors of the protein–protein interface.

Given a complex made up of *n* subunits *A*_1_, *A*_2_, ⋯, *A*_*n*_, the free energy of dissociation *Δ* *G*_DISS_ is defined as follows:

(6) ΔGDISS=−ΔGint−TΔSDISS.

*Δ* *G*_int_ is the binding energy of the subunits *A*_*i*_, *Δ* *S*_DISS_ is the entropy variation upon dissociation; unstable protein complexes correspond to *Δ* *G*_DISS_ < 0. *T* *Δ* *S*_DISS_ mostly contributes to *Δ* *G*_DISS_: it describes the loss of entropy (*T* *Δ* *S*_DISS_<0) of solvent molecules upon complex formation.

The binding energy of the subunits *Δ* *G*_int_ includes contributions from the following:

1. The solvation of the complex, comparing its value to that of single subunits *A*_*i*_;
2. The contact‐dependent (short‐range) interactions between chains;
3. The electrostatic (long‐range) interactions between chains.

Given the subunit interface, the solvation free energy gain upon formation of the interface *Δ* ^*i*^ *G* is calculated as a difference in total solvation energies of isolated and interfacing structures [42]. Negative values point to hydrophobic interfaces or positive protein affinity.

**Figure 2:**
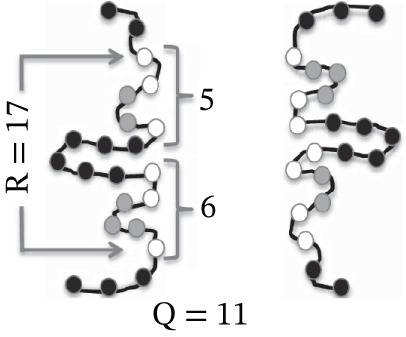
Protein–protein interface model. In white, the residues directly involved in the interface between different chains; in black, those excluded (outside the sequence trait directly involved in the interface or forming large loops within the interface sequence trait); and in gray, those forming small loops within the sequence trait participating in the interface but not directly involved in it.

Additionally, we computed two descriptors of the protein–protein interface for each chain [25] according to the interface model shown in Figure 2, where *R* is the length (in residues) of the sequence involved in the surface and *Q* is the number of residues involved in interchain contacts:

– The surface “roughness” *Q*/*R*: the lower *Q*/*R*, the rougher the interface;
– The interface amino acid range (IAR): it is defined as *R*/*N*, where *N* is the total number of residues in the chain.

## 3. Results and Discussion

### 3.1. Analysis of the Valorization Processes for Crustacean Shells

As outlined in Section 1, the biorefinery paradigm provides a general approach for the full valorization of crustacean waste. The application of the biorefinery paradigm becomes economically sustainable if high‐value‐added components are extracted: in the case of crustacean biorefinery, chitin and astaxanthin play a central role in the economic valorization of the whole process. Both components fuel a growing market for products meant for wellness and beauty.

**Figure 3:**
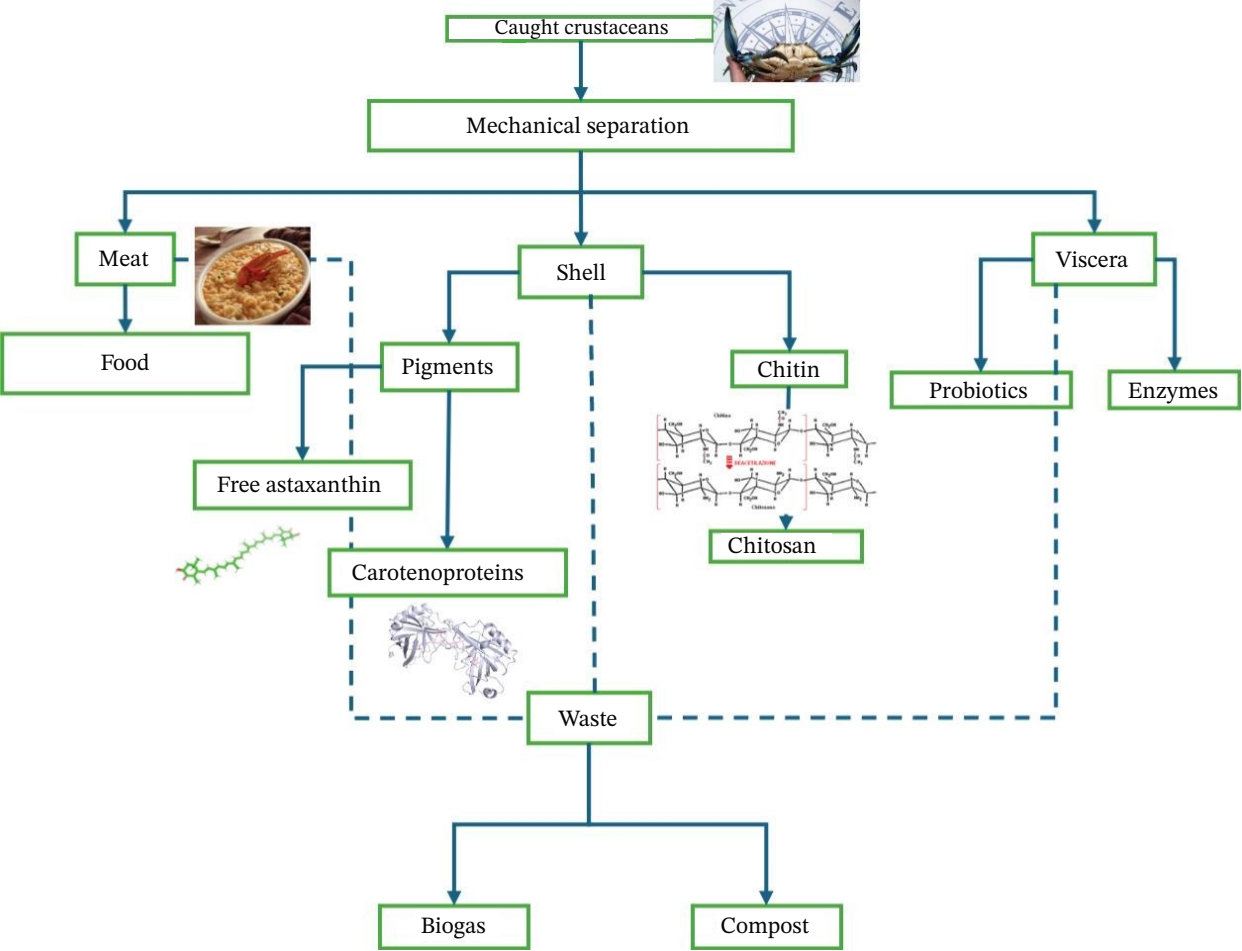
Biorefinery of crustacean fishery waste and by‐products.

In this context, the biorefinery of crustacean fisheries provides a wide spectrum of valuable products, as shown in Figure 3.

After the meat removal for food production, the crab and lobster shells (around 50% *w*/*w* [43]) are still rich in valuable compounds. Shell chitin has great potential as a biomaterial: the application of chitosan, produced by deacetylation of chitin, spans from the pharmaceutical and cosmetic sectors to applications in wastewater treatment, and emerging applications are driving a steeply growing market [44, 45]. Recent advances in chitin extraction and chitosan production have allowed a substantial reduction in the impact of traditional production methods [46,47].

Accordingly, lobster shell pigments hold considerable promise as functional compounds in the nutraceutical and cosmetic industries. In terms of economic value and market potential, they are comparable to chitin, equally driving a rapidly expanding global market. Notably, the primary pigment, astaxanthin, is projected to reach a global market value of $167.50 million by 2030, propelled by its growing demand as a dietary supplement for human consumption [48].

However, its applications are limited to dry or oil extracts due to its poor water solubility. The encapsulation of astaxanthin in nanoparticles can greatly improve its intestinal absorption and oral bioavailability [49].

Natural carriers of astaxanthin fall within the family of carotenoproteins, complexes of carotenoids and proteins widespread across living organisms, such as crustacyanin in decapods. Carotenoproteins exert different biological functions, from camouflage to photoprotection in different organisms. Table 2 shows the distribution of carotenoproteins and their features in invertebrates.

**Table 2:** Carotenoproteins in invertebrates.

Group/protein	Source	Protein structure	Carotenoid	Color effect	Biological function	Ref.
Crustacyanin (*α*, *β*)	Decapods	Lipocalins: *β* = heterodimer; *α* = oligomeric complex (8 × *β*)	Astaxanthin	Strong bathochromic shift (red → blue/green)	Camouflage, communication, UV protection, storage	[50]
Copepod carotenoprotein	Planktonic copepods	Simple proteins, often monomeric	Astaxanthin (esterified or free)	Bright red	UV protection, coloration for predation	[51]
*Daphnia* carotenoprotein	Cladocerans	Carotenoid‐binding proteins, not lipocalins	Astaxanthin, lutein	Red‐orange	UV protection, phenotypic response to predators	[52]
Isopod carotenoprotein	Marine and terrestrial isopods	Poorly characterized, simpler proteins	Astaxanthin, *β*‐carotene	Yellow‐orange	Camouflage, defense	[53]

Carotenoproteins in decapods are responsible for the bathochromic shifts of color in decapod shells [50]. For instance, crustacyanin in *H. gammarus* shell is a large complex comprising 16 molecules of astaxanthin (*α*‐crustacyanin), made up of eight repeats of *β*‐crustacyanin, a heterodimer containing two molecules of astaxanthin.

The astaxanthin complexation by carotenoproteins in decapods accomplishes different functions. The protein complex causes a shift in the astaxanthin absorption spectrum. This results in a change of color from the typical red‐orange of free astaxanthin to blue, green, or purple hues observed in certain crustacean species. This color change can be crucial for camouflage, mating displays, and species recognition [54].

Astaxanthin is a highly unsaturated molecule; this makes it susceptible to oxidation and degradation, especially when exposed to light and oxygen. Binding to crustacean crustacyanin provides protection for astaxanthin against photooxidation [55]; therefore, isolation, purification, and commercialization of astaxanthin complexed with crustacean crustacyanin would be an innovation in the pharmaceutical and cosmetic industries, since it could enhance astaxanthin shelf life and bioavailability. Recently, innovative extraction methods have also allowed further improvement in the sustainability of this product [56].

### 3.2. Results of the Computational Analysis

The application of the PCN method to the structure of carotenoproteins outlines crucial regions in the proteins responsible for dimerization and for the interaction with astaxanthin.

**Figure 4:**
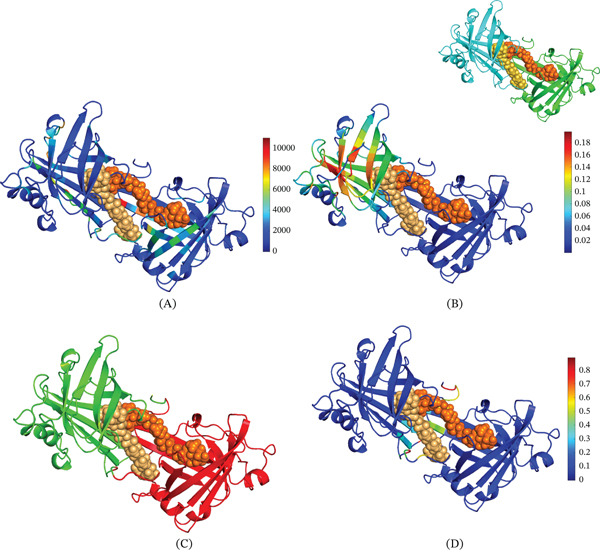
PCN results for *H. gammarus β*‐crustacyanin as heat maps. (A) Betweenness centrality. (B) Eigenvector centrality. (C) Two‐cluster partition. (D) Participation coefficient. The two astaxanthin molecules are represented as yellow and orange spheres. In the upper right side of the picture, *β*‐crustacyanin is colored according to chain (Chain A in green and Chain B in cyan).

Figure 4 reports the heat maps of the PCN results for the *β*‐crustacyanin of *H. gammarus*.

The betweenness centrality is well distributed and highlights key residues responsible for the interchain interactions and involved in the contacts with the astaxanthin molecules (Figure 4A). On the other hand, the eigenvector centrality is unevenly distributed, with higher values concentrated in Chain B (Figure 4B).

The partition into two clusters matches well with the two chains, which act as functional units in the protein structure (Figure 4C). The residues in the frontier region between the two chains are endowed with high values of the participation coefficient *P*, contributing to the molecular integrity and to the interactions with the astaxanthin molecules (compare Figure 4D with Figure 1A).

**Figure 5:**
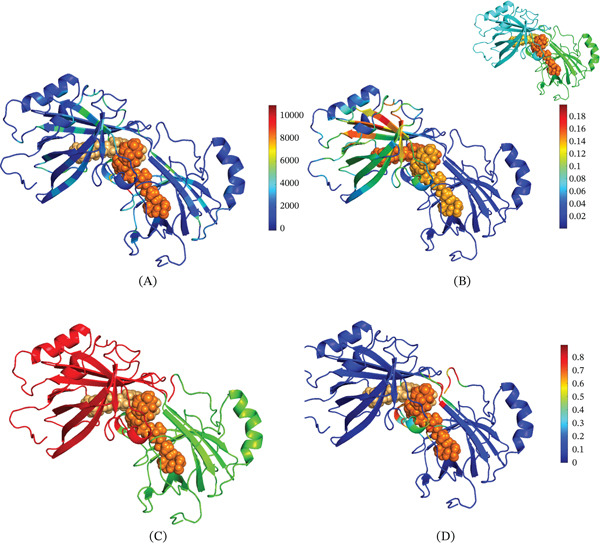
PCN results for the *C. sapidus* apolipoprotein D‐like protein as heat maps. (A) Betweenness centrality. (B) Eigenvector centrality. (C) Two‐cluster partition. (D) Participation coefficient. The two astaxanthin molecules are represented as yellow and orange spheres. In the upper right side of the picture, the apolipoprotein D‐like protein is colored according to chain (Chain A in green and Chain B in cyan).

Similarly, Figure 5 reports the heat maps of the PCN results for the apolipoprotein D‐like protein from *C. sapidus*.

As observed for the similar structure in *H. gammarus*, the betweenness centrality across the *C. sapidus* apolipoprotein appears quite regular across the molecule (Figure 5A); the highest values are for residues in contact with the two astaxanthin molecules (compare with Figure 1B).

Again, the eigenvector centrality (Figure 5B) is unevenly distributed across the molecule, as in the corresponding molecule in *H. gammarus*, but its distribution is less heterogeneous here.

Close to what was found in *H. gammarus β*‐crustacyanin, for the homologous molecule in *C. sapidus*, the two identified clusters match the two chains (Figure 5C); the highest values of the participation coefficient *P* correspond to the residues located at the interface between the two chains and partly to the residues in contact with the astaxanthin molecules (compare Figure 5D with Figure 1B).

**Figure 6:**
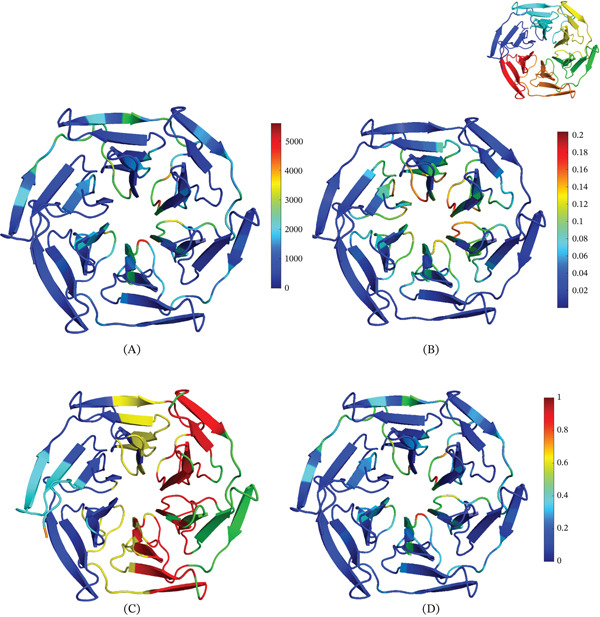
PCN results for the unbound Keap1, or kelch domain, as heat maps. (A) Betweenness centrality. (B) Eigenvector centrality. (C) Six‐cluster partition. (D) Participation coefficient. In the upper right side of the picture, the six domains of Keap1 are shown in different colors.

The above‐described results suggest that the biological function of these molecules (carriers of astaxanthin molecules) strictly determines their structures and functional dynamics. Indeed, noticeable residues are often those responsible for the interaction with the astaxanthin molecules. Figure 6 reports the PCN results for the unbound form of the Keap1‐DC domain.

The betweenness centrality distribution is quite even in the Keap1 molecular structure, with a prevalence in Blades I and II and on the narrowest side of the kelch domain (Figure 6A). The highest values of the eigenvector centrality, on the other hand, are mostly located at the kelch base, while the blades show smaller values (Figure 6B).

Figure 6C shows the cluster partition in six clusters, which do not match the six blades. Six clusters of different sizes are identified: a large cluster (in blue) comprising almost completely Blade VI, half of Blade I, and a part of Blade II; the red cluster is distributed across the molecule, comprising Blades III and V. Similarly, but smaller in size than the red one, the yellow cluster spreads over Blades II, V, and VI.

The cyan cluster covers a small part of Blade I, while the orange one is made up of only one residue, the N‐terminal end of Blade I. The participation coefficient distribution again highlights that the kelch base is the active region of the molecule (Figure 6D).

**Figure 7:**
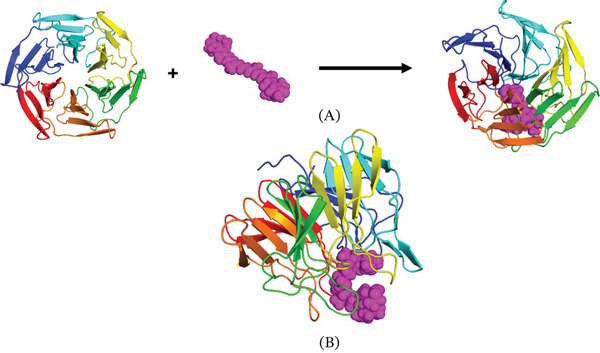
Molecular docking results for astaxanthin (ligand) on Keap1 (target). (A) The process of binding of the astaxanthin molecule (set in a straight standard conformation as input to the molecular docking software), resulting in a clear conformational change of both the receptor (Keap1) and astaxanthin, which is bent in the docked conformation. (B) A different perspective of the complex shows that the astaxanthin molecule in the bent conformation occupies the base of the kelch domain, capping it.

Figure 7 shows the result of the application of DiffDock‐L: the astaxanthin molecule is bent with respect to the straight initial conformation set as input, and it is located at the base of the kelch domain, mostly shifted toward Domain V.

The first striking evidence (Figure 7A,B) is that the astaxanthin molecule undergoes a conformational transition upon docking, as does the Keap1 molecule. It completely occupies the base of the kelch domain, possibly competing with Nrf2 (Figure 1F).

**Figure 8:**
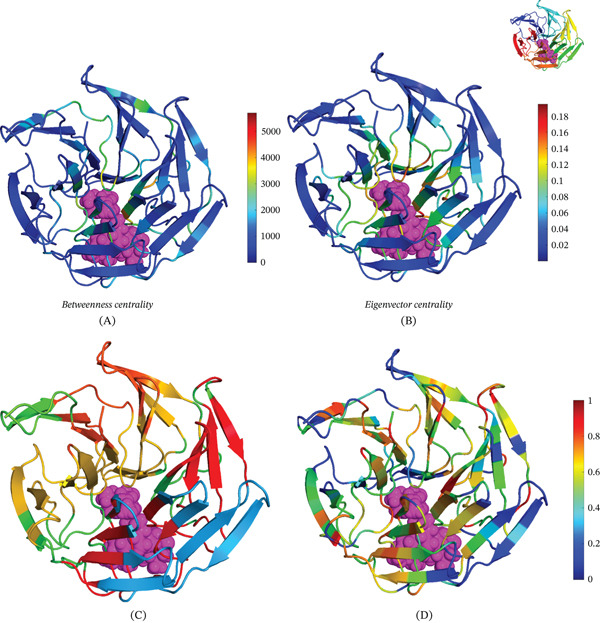
PCN results for Keap1 docked with astaxanthin as heat maps. (A) Betweenness centrality. (B) Eigenvector centrality. (C) Six‐cluster partition. (D) Participation coefficient. In the upper right side of the picture, the six domains of Keap1 are shown in different colors.

Figure 8 shows the results of the application of the PCN method to the bound form of Keap1 with one astaxanthin molecule.

The betweenness centrality distribution (Figure 8A) appears quite similar to that of the unbound form (Figure 6A), with a stronger prevalence of high values at the base of the kelch domain. On the other hand, the distribution of the eigenvector centrality (Figure 8B) shows that the highest values are mostly concentrated in the kelch base, in comparison with the distribution in the unbound form (Figure 6B).

The partition into six clusters (Figure 8C) in the bound form again does not match the domains but does so in a different way from the unbound form (Figure 6C): the two largest clusters (in cyan and red) enclose the region where the astaxanthin molecule binds, confirming the functional role of the interactions between the ligand and the Keap1 kelch domain in the complex. The participation coefficient map (Figure 8D) shows relevant residues in the intercluster communication, most falling at the kelch base.

**Figure 9:**
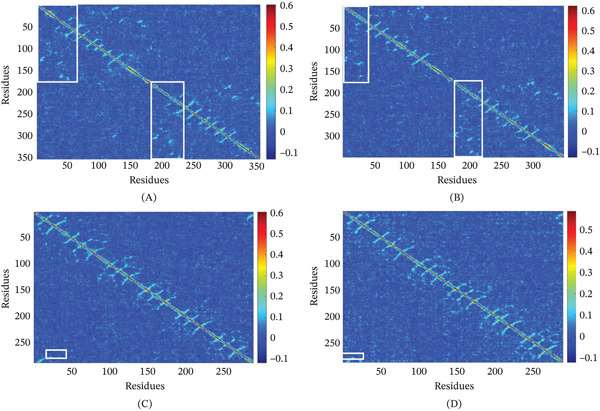
DCC maps from the molecular dynamics simulations of the analyzed protein structures. (A) *H. gammarus*. (B) *C. sapidus*. (C) Keap1, unbound form. (D) Keap1, bound form, in complex with astaxanthin. For the two carotenoproteins, the white rectangles highlight the correlations of the N‐terminal region of Chain B with the rest of the chain. For the Keap1 forms, the white rectangles highlight the correlations of the C‐terminal region with the N‐terminal region, which form Domain I.

The DCC maps for the two carotenoproteins are shown in Figure 9A,B.

The two DCC maps for the two carotenoproteins (Figure 9A,B) display highly similar patterns, with noticeable correlations (greenish spots) mainly located within the chains. The regions highlighted by the white rectangles are shown for comparison: in both carotenoproteins, these areas of Chain B correspond to the first two *β*‐sheet strands in the N‐terminal region.

Similarly, the corresponding DCC heat maps for the Keap1 forms (unbound and bound to astaxanthin) are reported in Figure 9C,D. The white rectangles highlight the interactions between the first two N‐terminal strands and the C‐terminal region, which together form Domain I. In addition, diffuse interactions (small off‐diagonal spots) are observed, but they do not show significant changes between the free and astaxanthin‐complexed kelch domain of Keap1.

Table 3 reports the stability and chemophysical parameters of the interface in carotenoproteins.

**Table 3:** Descriptors of the interface between chains in carotenoproteins.

Chain	*n*_*R* *E* *S*_	*Δ* *G*(kcal/mol)	${n_{RES}}^{\mathrm{int}}$	*Q*/*R*	IAR	*Δ* *G* ^int^(kcal/mol)
*Homarus gammarus*						
A	180	−153.8	13	0.11	0.68	−11.15
B	174	−163.5	12	0.10	0.70	−13.39
*Δ* *G*_DISS_ = −1.7 kcal/mol *T* *Δ* *S*_DISS_ = 12.2 kcal/mol *Δ* ^*i*^ *G* = −7.0 kcal/mol						
*Callinectes sapidus*						
A	174	−137.1	13	0.11	0.71	−9.73
B	174	−142.7	17	0.14	0.70	−6.64
*Δ* *G*_DISS_ = −3.2 kcal/mol *T* *Δ* *S*_DISS_ = 12.0 kcal/mol *Δ* ^*i*^ *G* = −5.1 kcal/mol						

For both carotenoproteins, Chain B is more stable than Chain A. Chain B participates with fewer residues than does *H. gammarus* Chain A, while its residues participating in the interface are more numerous than those in *C. sapidus* Chain A. For both proteins, the interfaces are rough (*Q*/*R* around 0.1), and the portion of the sequence involved in the interface is wide (around 70%). This confirms the high specialization of the molecular structure in building up an efficient and stable carrier for astaxanthin by means of the interchain interface.

As for the energy of the subunits, for the *β*‐crustacyanin of *H. gammarus*, Chain B is slightly more stable than Chain A; the inverse happens in the homologous apolipoprotein D‐like protein of *C. sapidus*. Both forms are slightly unstable (*Δ* *G*_DISS_ < 0), with the form from *H. gammarus* more stable than that from *C. sapidus.* The two complexes are largely stabilized by an increase in entropy (*T* *Δ* *S*_DISS_ > 0). Finally, the negative values of *Δ* ^*i*^ *G* indicate that the interface between the two chains in both complexes is hydrophobic (positive protein affinity).

Table 4 reports the protein stability for all the Keap1 forms (computation was applied to the final frame after the relaxation over 1 ns): the bound forms appear very similar in stability, confirming the possible role of astaxanthin as a competitive inhibitor of the binding to the Nrf2 peptide.

**Table 4:** Protein stability of Keap1 forms.

Keap1 form	*Δ* *G*_unfolding	RMSD (Å)
Unbound	14.474	—
Kelch–Nrf2	14.201	1.147
Kelch–AXT	14.457	0.930

Additionally, the RMSD value for the Nrf2 apo form, with respect to the bound form, is slightly larger than that for the Keap1–AXT complex, further promoting the latter as a possible complex if astaxanthin molecules are present in the Keap1 microenvironment.

## 4. Conclusions

In this study, we analyze the structural properties of two crustacean carotenoproteins that complex astaxanthin. We also demonstrate in silico the biological activity of astaxanthin as an antioxidant agent, targeting the human Keap1 kelch domain. These findings provide molecular‐level evidence of how seafood processing by‐products, often considered environmental burdens, can be transformed into sustainable sources of bioactive molecules.

We hereby produce a computational model for the *C. sapidus* apolipoprotein D‐like protein, homologous to *H. gammarus* crustacyanin.

The stable carotenoprotein complex protects astaxanthin from photooxidation; thus, this complex represents a new, more stable source of astaxanthin, with improved pharmacokinetic and possibly pharmacodynamic properties.

Therefore, we hereby provide insight into an innovative nutraceutical formulation of astaxanthin complexed with crustacean carotenoproteins isolated from shell waste.

Currently, the European Commission considers astaxanthin to be a food dye, but in Italy, hundreds of nutraceuticals for human consumption contain astaxanthin as one of the active principles (https://www.salute.gov.it/new/it/tema/alimenti-fini-medici-speciali-ed-integratori/registro-degli-integratori-alimentari/), usually extracted from the microalga *Haematococcus pluvialis* [57]. Therefore, carotenoproteins complexing astaxanthin would be not only an alternative and sustainable source of astaxanthin but also a complex that serves as an alternative food supplement, improving the safety and efficacy of astaxanthin compared to the free form [58].

We also identify the Keap1‐DC domain as a molecular target for the astaxanthin antioxidant agent, competing with Nrf2 binding and promoting its induction, highlighting a pathway of considerable interest in oncology.

All in all, our results highlight the relevance of the valorization of crustacean shell waste to foster circular economy principles, contributing simultaneously to waste reduction, environmental protection, and innovation in food and health sciences. The use of invasive species such as *C. sapidus* in the Mediterranean Sea area further underscores the dual benefit of mitigating their ecological impact while unlocking the biotechnological potential of these fishery resources.

While additional experimental validation and translational studies are needed, this work paves the way for the development of seafood‐derived functional ingredients and therapeutic products. In doing so, it reinforces the concept that marine waste and by‐products represent a valuable reservoir for sustainable biorefinery processes, bridging food science, biotechnology, and human health.

## Funding

Funding was provided by the Next Generation EU Fund, Hub Rome Technopole (Spoke 2). Open access publishing was facilitated by Università Campus Bio‐Medico di Roma as part of the Wiley‐CRUI‐CARE agreement.

## Conflicts of Interest

The authors declare no conflicts of interest.

## Data Availability

Data supporting the findings of this study are available on request from the corresponding authors. Data are not publicly available due to privacy or ethical restrictions.
